# ‘The Healthy Migrant Effect’ for Mental Health in England: Propensity-score Matched Analysis Using the EMPIRIC Survey

**DOI:** 10.1007/s10903-017-0570-z

**Published:** 2017-04-07

**Authors:** Amrit Dhadda, Giles Greene

**Affiliations:** Institute of Primary Care & Public Health, Cardiff University School of Medicine, 3rd Floor Neuadd Meirionnydd, Heath Park, Cardiff, CF14 4YS UK

**Keywords:** Immigrants, Healthy migrant effect, Mental health, UK

## Abstract

Evidence has demonstrated that immigrants have a mental health advantage over the indigenous population of developed countries. However, much of the evidence-base demonstrating this mental health advantage is susceptible to confounding and inadequate adjustment across immigrant and non-immigrant groups preventing a rigorous assessment of a ’healthy migrant effect’. To compare the risk of common mental disorders in the immigrant population compared to the non-immigrant population in ethnic minority groups in England. A propensity-score matched analysis was carried out to adequately balance immigrant and non-immigrant groups for known confounders using the EMPIRIC national survey of Black-Caribbean, Indian, Pakistani and Bangladeshi groups. The mental health of participants was assessed using the validated Revised Clinical Interview Schedule tool. Immigrant participants were significantly less likely to have a common mental disorder than non-immigrant participants; OR = 0.47, (95% CI 0.40, 0.56). The results from this study demonstrate that a mental health advantage exists in ethnic minority immigrants compared to non-immigrants when balancing the two groups for confounding factors. This may be due to immigrants possessing certain personality traits, such as "psychological hardiness", that the migration process may select for.

## Introduction

A growing body of evidence has demonstrated the epidemiological paradox that immigrants have better overall health than the indigenous population of developed countries, named ‘the healthy migrant effect’, which describes both a physical and mental health advantage [1–4]. Despite the growing body of evidence demonstrating this ‘healthy migrant effect’ in immigrants, there is limited up-to-date literature comparing immigrant and non-immigrant minorities i.e. second or third generation descendants.

A four year cohort study looking at second generation immigrants in Sweden demonstrated a greater rate of hospitalisation due to psychotic, affective, neurotic and personality disorders than the majority population [5]. Another study looking at mood, anxiety and personality disorders amongst first and second generation immigrants in the United States demonstrated the ‘healthy migrant effect’ when comparing immigrants with the indigenous population, but there was a higher prevalence of mental health conditions in second generation migrants [6].

A number of potential reasons may exist for a potential mental health advantage in immigrants compared to non-immigrant minorities; one important explanation theorises that certain selection factors may favour immigrants, such as the drive to take chances and negotiate difficult circumstances. These personality attributes may provide a more robust coping mechanism when dealing with some of the adversity associated with the minority status [7, 8]. Another theory describes how acculturative stressors affect non-immigrant minorities differently; this includes asymmetric acculturation from being born in different countries and attempting to integrate in their resident country and facing restrictive processes due to discrimination and a weak position within society [7–10].

However, much of the evidence-base demonstrating a greater prevalence of mental health conditions in non-immigrant minorities is susceptible to confounding and inadequate adjustment across immigrant and non-immigrant groups, preventing a rigorous assessment of mental health between the two groups [11, 12]. Furthermore, there has been a lack of exploration of migrant health in a UK setting; much of the available evidence demonstrating a mental health advantage in immigrants has focused on North American and other European populations [2, 13].

The UK has become a very multicultural and ethnically diverse society, with the non-UK born population quadrupling from 1.9 million (4.5% of the population) in 1951 to 7.5 million (13% of the population) in 2011 [14]. There is a crucial knowledge gap in understanding the impact of mental health conditions affecting the diverse immigrant and minority populations in the UK. A better understanding in this area can help safeguard the immigrant and ethnic minority populations and if necessary address areas for which interventions and education can be better directed towards.

Using the cross-sectional Ethnic Minority Psychiatric Rates in the Community (EMPIRIC) survey the aim of this study is to compare the risk of common mental disorders, as a global measure, in immigrants compared to non-immigrants in ethnic minorities in the UK.

## Method

### Sample

This study utilised data collated for the EMPIRIC national population survey in 2000 (n = 4281). The EMPIRIC study is a cross-sectional survey carried out to assess the prevalence of common mental disorders in different ethnic groups including White, Black-Caribbean, Bangladeshi, Indian and Pakistani groups in England. Participants from the ethnic minority groups were obtained from the 1999 Health Survey for England [15]. White participants were taken from the 1998 Health Survey for England [16]; the general population questionnaire and measures being very similar to the ethnic minority 1999 Survey. The EMPIRIC study excluded the 8% of participants who did not want to be re-contacted and selected their sample from participants aged 16–74. Data from the EMPIRIC study were accessed through the UK data archive and this study adhered to the conditions to the agreements of use. Ethical approval for EMPIRIC was obtained from the North Thames Multi-centre Research Ethics Committee (MREC) and ratified by all Local Research Ethics Committees (LRECs) in England.

The EMPIRIC study utilised questions from existing validated instruments such as SF-12 physical and mental health summary scales [17], and the revised clinical interview schedule (CIS-R) [18]. Social support was measured using tools such as Whitehall II study of British civil servant [19], and social functioning questionnaire [20]. Full details of the EMPIRIC survey including the measures recorded are described in greater detail [21, 22].

### Measures

An assessment for the presence of a common mental disorder was undertaken using the CIS-R, a widely used tool in the UK [23, 24]. The CIS-R is considered by many researchers to be the most valid and reliable tool for assessing common mental disorders, particularly anxiety and depressive disorders, according to ICD-10 and DSM-IV diagnostic criteria [25, 26]. The CIS-R enquires about the presence and severity of 14 non-psychotic symptoms in the week before the interview. These cover somatic complaints including fatigue, problems with memory and/or concentration and sleep disturbance, and emotional symptoms such as depressed mood and thoughts, anxiety and obsessional thoughts. A CIS-R score of 12 or more has been shown to be a suitable case threshold in determining the presence of a common mental disorder, and was used in the present study [21]. The EMPIRIC survey documented whether participants were immigrants allowing the present study to define the risk of common mental disorders by immigrant status.

The present study controlled for several factors that have been shown to affect the risk of developing common mental disorders [27]. The participants gender and age were recorded, the latter being categorised into three bands; 16–34, 35–54 and 55–74 years old. Social class was based on the occupation of the head of the household and classified according to the Registrar General’s classification using the six-point scale; class I (professional), II (managerial/technical), IIIa (skilled, non-manual), IIIb (skilled, manual), IV (semi-skilled), V (unskilled) [28]. Participants’ marital status was recorded into five categories; single, married, separated, divorced and widowed. Educational achievement was classified into NVQ4/5/degree equivalent, higher education below degree, NVQ3/A-Level equivalent, NVQ2/O-Level equivalent, NVQ1/CSE equivalent, foreign/other educational achievement or no qualifications. The religious or spiritual beliefs of participants were recorded into religious, spiritual or none. Ethnic origin was self-defined using the census classificatory system [29]; White, Black-Caribbean, Indian, Pakistani and Bangladeshi. The short form 12-item survey (SF-12), derived from the longer SF-36, measures the participants’ physical health and has been demonstrated to be reliable in clinical and population-based applications worldwide [17, 30]. The participant’s answers are computed to give a score from 0 to 100, where 0 indicates the lowest level of health and 100 indicates the highest level of health [17]. In order to get a notion of the social interaction and potential adversity faced by participants this study utilised information on whether there was regular contact with friends (yes/no), number of people participants were close to (0–60) and whether they had been physically attacked in the last 12 months (yes/no). Chronic social strain was measured by questions from the Whitehall II study [19]; a five-point Likert scale was used for domains considering problems with relatives, financial problems, housing problems, and difficulties in the local neighbourhood. These were combined to give an overall score (on a scale from 0 to 5), where a higher score indicated more frequent or greater social strain [20].

### Statistical Analysis

Baseline characteristics of the sample were calculated using proportions and medians as appropriate and were compared between the immigrant and non-immigrant groups using χ^2^ test for categorical variables and Mann–Whitney U test for continuous variables. We determined a propensity score for common mental disorders according to immigration status using a logistic regression model [31, 32].

Propensity-score matching is a statistical principle that attempts to overcome a key limitation of making causal inferences from observational study designs, where no systematic methods have been used to maintain control groups. Propensity-score matching involves pairing exposure (or treatment) and comparison units in terms of their observable characteristics. This produces two groups that are matched according to a set of measured covariates. Propensity-score matching methods are being increasingly used in medical research to provide unbiased estimates from observational data where assignment to treatment or exposures is typically not random [33, 34].

The covariates entered into the propensity score are described in detail earlier and include socio-demographic factors (gender, age, social class, education attainment, ethnic background and religious or spiritual beliefs), physical health status (SF-12 general health score) and social measures (chronic social strain score, number of people close to, regular contact with friends and whether physically attacked in the previous 12 months). Immigrants were matched to non-immigrants on the logit of the propensity score by using calipers of width equal to 0.2 of the standard deviation of the logit of the propensity score [35]. A matching ratio of 1:1 was used [36]. The matched characteristics of the cohort represent reweighted figures on the propensity score [37].

We estimated standardised differences for all covariates before and after matching, with a standardised difference of 20% or more considered indicative of imbalance [34, 37]. The presence of common mental disorders between immigrant and non-immigrant participants were compared after matching using methods appropriate for the analysis of matched data to estimate the effect of immigration status and its significance. We determined the odds ratio of common mental disorders using a logistic regression model. We determined three models of common mental disorder risk development according to immigrant status; unadjusted model (model 1), adjusted model for covariates (model 2) and for covariates entered into the propensity score for matching both immigrants and non-immigrants (model 3).

To assess the sensitivity of the results we tested the influence of unmeasured confounders, which if neglected may influence the participation probabilities, i.e. two individuals with the same observed covariates may have differing chances of allocation to treatment/exposure group. In a study free of hidden bias, participation probability will be based solely on their observed measurements. However, with hidden bias an unmeasured factor may be driving allocation to treatment and thus moderating any perceived treatment effect. Using the Mantel–Haentzel statistic using the STATA programme *mhbounds* [38], we calculated the test statistic for the risk of screening positive for a common mental disorder for differing degrees of assignment due to unobserved factors. All analyses were performed in STATA version 13 for Windows [39].

## Results

### Cohort Characteristics

Of the 4281 members taking part in the EMPIRIC survey, 2069 were immigrants and 2212 were non-immigrants (UK-born). All participants were included in this present study. Of the 2069 immigrants, 350 (16.9%) were assessed as having a common mental disorder, compared to 385 (17.4%) of the non-immigrants.

Table 1 shows the characteristics of the immigrant and non-immigrant participants before and after matching. Before matching immigrants in the study were less likely to be in the younger 16–34 age band (27.07 vs. 49.14%, P < 0.001), in social classes I-III (31.12 vs. 47.42%, P < 0.001), in the White ethnic group (0.10 vs. 37.75%, P < 0.001) and had a lower median SF-12 physical health score (50.50 vs.54.21, P < 0.001). They were also more likely to be married (73.22 vs. 44.67%, P < 0.001), have religious beliefs (86.52 vs. 52.98%, P < 0.001) and be in the Indian, Pakistani or Bangladeshi ethnic groups (71.10 vs. 24.68%, P < 0.001).

**Table 1:** Comparison of demographic characteristics of participants of the EMPIRIC study before and after matching by immigration status including the significance of covariates

Characteristics	Before matching (n = 4281)					After matching (n = 3224)			
	Non-immigrants (n = 2212)		Immigrants (n = 2069)	Standardised difference	Sig (<0.05)	Non-immigrants (n = 1612)	Immigrants (n = 1612)	Standardised difference	Sig (<0.05)
Gender (%)					ns				ns
Male	985 (44.53)		956 (46.21)	0.09		625 (38.77)	760 (47.15)	0.17	
Female	1227 (55.47)		1113 (53.79)	0.09		987 (61.23)	852 (52.85)	0.17	
Missing	0 (0.00)		0 (0.00)						
Banded age (%)					<0.001				ns
16 to 34 years	1087 (49.14)		560 (27.07)	0.50		406 (25.19)	443 (27.48)	0.05	
35 to 54 years	809 (36.57)		913 (44.13)	0.25		884 (54.84)	736 (45.66)	0.18	
55 to 74 years	316 (14.29)		596 (28.81)	0.29		322 (19.98)	433 (26.86)	0.16	
Missing	0 (0.00)		0 (0.00)						
Social class^a^ (%)					<0.001				ns
I	148 (6.69)		115 (5.56)	0.02		59 (3.66)	94 (5.83)	0.10	
II	621 (28.07)		346 (16.72)	0.28		304 (18.86)	260 (16.13)	0.07	
III non manual	280 (12.66)		183 (8.84)	0.14		67 (4.16)	149 (9.24)	0.21	
III manual	614 (27.76)		636 (30.74)	0.06		417 (25.87)	498 (30.89)	0.11	
IV	380 (17.18)		524 (25.33)	0.23		564 (34.99)	426 (26.43)	0.19	
V	77 (3.48)		100 (4.83)	0.06		81 (5.02)	79 (4.90)	0.01	
Other/never worked	67 (3.03)		125 (6.04)	0.13		120 (7.44)	106 (6.58)	0.03	
Missing	25 (1.13)		40 (1.93)						
Legal marital status (%)					<0.001				ns
Married/cohabiting	988 (44.67)		1515 (73.22)	0.66		1240 (76.92)	1217 (75.50)	0.03	
Single	865 (39.10)		240 (11.60)	0.84		154 (9.55)	169 (10.48)	0.03	
Separated	71 (3.21)		101 (4.88)	0.14		33 (2.05)	77 (4.78)	0.15	
Divorced	123 (5.56)		92 (4.45)	0.03		109 (6.76)	75 (4.65)	0.09	
Widowed	50 (2.26)		105 (5.07)	0.11		76 (4.71)	74 (4.59)	0.01	
Missing	115 (5.20)		16 (0.77)						
Highest qualification (%)					<0.001				ns
NVQ4-5/degree or equiv	294 (13.29)		272 (13.15)	0.00		221 (13.71)	216 (13.40)	0.05	
Higher ed below degree	263 (11.89)		148 (7.15)	0.19		92 (5.71)	108 (6.70)	0.11	
NVQ3/A-level equiv	306 (13.83)		138 (6.67)	0.30		69 (4.28)	100 (6.20)	0.02	
NVQ2/O-level equiv	605 (27.35)		309 (14.93)	0.34		220 (13.65)	239 (14.83)	0.04	
NVQ1/CSE or other equiv	114 (5.15)		67 (3.24)	0.13		74 (4.59)	52 (3.23)	0.02	
Foreign qual or other	90 (2.71)		103 (4.98)	0.11		59 (3.66)	77 (4.78)	0.06	
No qualifications	455 (20.57)		1015 (49.06)	0.63		877 (54.40)	820 (50.87)	0.07	
Missing	115 (5.20)		17 (0.82)						
Religious or spiritual beliefs (%)					<0.001				ns
Religious	1172 (52.98)		1790 (86.52)	0.82		1327 (82.32)	1423 (88.28)	0.17	
Spiritual	404 (18.26)		156 (7.54)	0.32		162 (10.05)	120 (7.44)	0.09	
Neither	624 (28.21)		120 (5.80)	0.69		123 (7.63)	69 (4.28)	0.14	
Missing	12 (0.54)		3 (0.14)						
Ethnic group^b^ (%)					<0.001				ns
White	835 (37.75)		2 (0.10)	1.39		4 (0.25)	2 (0.12)	0.03	
Black-caribbean	363 (16.41)		331 (16.00)	0.04		253 (15.69)	305 (18.92)	0.09	
Indian	180 (8.14)		463 (22.38)	0.44		407 (25.25)	428 (26.55)	0.03	
Pakistani	262 (11.84)		462 (22.33)	0.26		353 (21.90)	404 (25.06)	0.08	
Bangladeshi	104 (4.70)		546 (26.39)	0.68		595 (36.91)	473 (29.34)	0.16	
Missing	468 (22.16)		265 (12.81)						
Chronic strain score (%)					<0.001				ns
0	542 (24.50)		484 (23.39)	0.05		399 (24.75)	363 (22.52)	0.05	
1	744 (33.63)		553 (26.73)	0.17		348 (21.59)	421 (26.12)	0.11	
2	425 (19.21)		404 (19.53)	0.05		340 (21.09)	325 (20.16)	0.02	
3	279 (12.61)		326 (15.76)	0.08		287 (17.80)	263 (16.32)	0.04	
4	149 (6.74)		240 (11.60)	0.10		212 (13.15)	200 (12.41)	0.02	
5	56 (2.53)		52 (2.51)	0.02		26 (1.61)	40 (2.48)	0.06	
Missing	17 (0.77)		10 (0.48)						
Physically attacked in last year (%)					<0.01				ns
Yes	124 (5.61)		71 (3.43)	0.09		45 (2.79)	55 (3.41)	0.04	
No	2086 (94.30)		1998 (96.57)	0.09		1567 (97.21)	1557 (96.59)	0.04	
Missing	2 (0.09)		0 (0.00)						
Regular contact with friends (%)					<0.001				ns
Yes	2024 (91.50)		1739 (84.05)	0.23		1308 (81.14)	1351 (83.81)	0.07	
No	188 (8.50)		330 (15.95)	0.23		304 (18.86)	261 (16.19)	0.07	
Missing	0 (0.00)		0 (0.00)						
Median no. of people close to (IQR)*	5 (3)		5 (8)	0.17	<0.001	4 (5)	5 (8)	0.11	<0.05
Median SF-12 Physical health score (IQR)*	54.21 (7.40)		50.59 (13.45)	0.44	<0.001	51.28 (15.73)	50.41 (13.45)	0.07	ns

^a^ Registrar General’s social classification scheme (28)

^b^ Self-defined using the Census classification system (29)

^*^ Continuous outcome described as median and (interquartile range)

### Matching

1612 immigrant participants (77.9%) were successfully matched to non-immigrant participants. After matching, the absolute standardised differences were less than 20% for virtually all variables entered into the propensity score, indicating an adequate match [31]. Only social class III (non-manual) had a higher standardised difference, at 21% (4.16% non-immigrants, 9.24% immigrants). 457 immigrant participants could not be matched to a suitable control. Compared to those matched, the unmatched immigrant participants were similar in terms of age and gender but were less likely to be religious, (80.3 vs. 88.3%), married/cohabiting (65.2 vs. 75.5%) and more likely to be in social class II (18.8 vs. 16.1%). Fewer unmatched immigrant participants were assessed as having a common mental disorder; not matched 200 (16.8%) *v* matched 535 (17.3%).

### Outcomes After Matching

In the reweighted matched group containing 3224 participants, 766 (23.8%) were assessed as having a common mental disorder. Table 2 outlines the unadjusted, adjusted and propensity matched models for risk of having a common mental disorder according to immigration status. In the propensity-score matched analysis (model 3) immigrant participants were significantly less likely to have a common mental disorder than non-immigrant participants; odds ratio (OR) = 0.37, 95% confidence interval (95% CI) 0.29 to 0.46. Even in the unadjusted and covariate adjusted model, being an immigrant was associated with a lower risk of developing a common mental disorder, however the effect was not significant in these models; OR = 0.96 (95% CI 0.82, 1.13), OR = 0.76 (95% CI 0.56, 1.04) respectively.

**Table 2:** Odds ratio (95% confidence intervals) for immigrants developing a common mental disorder (≥12 on CIS-R score) in different models of analysis

	Model 1		Model 2		Model 3		Model 4	
	Odds ratio	95% CI	Odds ratio	95% CI	Odds ratio	95% CI	Odds ratio	95% CI
Immigration status								
Non-immigrant	Ref		Ref		Ref		Ref	
Immigrant	0.96	0.82, 1.13	0.76	0.56, 1.04	0.37	0.29, 0.46	0.60	0.45, 0.80
Gender								
Male			Ref		Ref		Ref	
Female			1.52	1.22, 1.89	1.32	1.04, 1.68	1.39	1.11, 1.75
Banded age								
16 to 34			Ref		Ref		Ref	
35 to 54			1.48	1.10, 1.98	3.04	2.17, 4.25	1.73	1.27, 2.37
55 to 74			1.14	0.77, 1.69	4.07	2.64, 6.28	1.34	0.90, 1.99
Social class								
I			Ref		Ref		Ref	
II			1.59	0.92, 2.75	3.50	1.58, 7.77	1.40	0.80, 2.47
III non manual			1.13	0.62, 2.07	2.09	0.88, 4.96	0.78	0.41, 1.49
III manual			1.19	0.68, 2.09	3.01	1.37, 6.65	1.15	0.66, 2.02
IV			1.26	0.71, 2.25	3.45	1.54, 7.73	1.88	1.07, 3.32
V			1.22	0.59, 2.52	6.51	2.64, 16.01	2.05	1.05, 4.01
Other/never worked			1.36	0.67, 2.57	6.53	2.68, 15.90	1.55	0.78, 3.06
Marital status								
Single			Ref		Ref		Ref	
Married			0.71	0.52, 0.97	0.41	0.27, 0.63	0.64	0.43, 0.94
Separated			0.89	0.52, 1.52	0.49	0.26, 0.94	0.57	0.31, 1.03
Divorced			0.59	0.35, 0.99	1.41	0.80, 2.94	0.64	0.34, 1.21
Widowed			0.50	0.26, 0.98	0.13	0.06, 0.29	0.29	0.15, 0.56
Physical health score			0.94	0.93, 0.95	0.91	0.90, 0.92	0.92	0.91, 0.93
Chronic strain score								
0			Ref		Ref		Ref	
1			2.24	1.55, 3.24	5.19	3.36, 8.00	3.62	2.47, 5.29
2			3.72	2.55, 5.43	9.85	6.39, 15.19	4.42	2.97, 6.58
3			4.70	3.17, 6.96	18.51	11.78, 29.10	6.47	4.28, 9.77
4			7.58	4.96, 11.59	11.85	7.40, 18.97	8.65	5.63, 13.29
5			13.37	7.46, 23.96	33.17	15.13, 72.72	14.31	7.16, 28.58
Education								
NVQ4-5 degree or equiv			Ref		Ref		Ref	
Higher Ed below degree			0.79	0.49, 1.28	0.56	0.29, 1.09	0.69	0.38, 1.25
NVQ3/GCE a level equivalent			1.14	0.73, 1.78	1.82	1.02, 3.22	1.09	0.63, 1.89
NVQ2/GCE o level equivalent			1.24	0.84, 1.83	2.52	1.62, 3.92	1.35	0.86, 2.10
NVQ1/CSE other grade equivalent			1.34	0.75, 2.38	0.58	0.28, 1.18	0.85	0.43, 1.68
Foreign/other			1.04	0.56, 1.91	1.64	0.86, 3.11	1.72	0.99, 3.01
No qualification			0.79	0.53, 1.18	0.60	0.38, 0.95	0.68	0.44, 1.05
Religious or spiritual belief								
Religious			Ref		Ref		Ref	
Spiritual			1.35	0.98, 1.87	0.97	0.63, 1.50	1.06	0.68, 1.65
Neither religious or spiritual			1.18	0.84, 1.65	2.02	1.28, 3.19	1.21	0.72, 2.02
Ethnic group								
White			Ref		Ref		Ref	
Black – Caribbean			1.02	0.70, 1.49	0.34	0.03, 3.28	0.95	0.62, 1.48
Indian			1.63	1.08, 2.45	1.42	0.15, 13.90	2.37	1.54, 3.64
Pakistani			1.69	1.11, 2.57	1.29	0.13, 12.82	2.52	1.64, 3.89
Bangladeshi			0.79	0.48, 1.32	0.75	0.08, 7.46	1.13	0.71, 1.80
Physically attacked in the last year								
Yes			Ref		Ref		Ref	
No			0.36	0.24, 0.55	0.51	0.29, 0.91	0.45	0.27, 0.73
Regular contact with friends								
Yes			Ref		Ref		Ref	
No			1.07	0.79, 1.46	0.59	0.43, 0.81	1.21	0.89, 1.64

Regression tables for the 4 models, Model 1 = unadjusted migration status, Model 2 = covariate adjusted, Model 3 = prosperity score matched sample matched on ethnic grouping, Model 4 = propensity score matched sample not matched on ethnic grouping

### Excluding Ethnic Grouping From the Propensity-score Matching

The EMPIRIC dataset contains only 837 (19.55%) participants in the White ethnic group and of these only 2 (0.24%) were immigrants. By including ethnic group into the propensity score we removed all but six of the White ethnic group participants from the matched analysis (2 immigrants, 4 non-immigrants), therefore the main analysis assesses the effect of immigration status mainly in Black-Caribbean, Indian, Pakistani and Bangladeshi groups. We therefore carried out an additional analysis, model 4, to exclude ethnic group from the propensity score to include a better pool of White non-immigrants to more accurately reflect the UK indigenous population. In this analysis, in the matched group 711 (38.3%) of non-immigrants were in the White ethnic group compared to the 4 (0.25%) in the main analysis and the 835 (37.75%) in the baseline sample. In this model immigrants were still significantly less likely to have a common mental disorder compared to non-immigrants (OR = 0.60, 95% CI 0.45, 0.80).

### The Effect of Unmeasured Confounders

Under the assumption of hidden bias, the test result gives similar results in confirming the lower risk of common mental disorders in immigrants. The results demonstrated that one would have to increase the odds of differential assignment due to unobserved factors by 55% in order to achieve a significant change and an overestimation of the effect. Therefore, we may suggest that the effects observed are resilient to overestimation or positive selection bias.

## Discussion

### ‘The Healthy Migrant Effect’ for Mental Health in England

The present study is based on a population of White, Black-Caribbean, Indian, Pakistani and Bangladeshi ethnic groups that were recruited as part of the EMPIRIC study to assess the prevalence of common mental disorders between different ethnic groups. The propensity-matched analysis sample assessed 766 (23.8%) as having a common mental disorder which is a similar proportion as the current UK population [40]. In the propensity matched analysis immigrants were significantly associated with a lower risk of common mental disorders than non-immigrants. By undertaking propensity-score matching we were able to balance both immigrant and non-immigrant groups for a number of established risk factors for poor mental health. In comparison the unadjusted and covariate adjusted model showed no significance between the immigrant and non-immigrant groups.

The EMPIRIC dataset contains only 837 (19.55%) participants in the White ethnic group and of these only 2 (0.24%) were immigrants. This analysis therefore demonstrated a mental health advantage in immigrants compared to non-immigrants mainly in the Black-Caribbean, Indian, Pakistani and Bangladeshi ethnic groups. Possible reasons for why immigrants may demonstrate better mental health than further generation migrants may be due to acculturative stressors may contribute to poorer mental health than new immigrants [6, 9, 10], other social causation factors may play a role, such as restricted opportunities in the face of higher aspirations [13, 41].

This study found evidence for a mental health advantage in ethnic minority immigrants in the UK and supports the evidence that has documented this effect in other developed countries such as America and Canada [1–4, 13]. The propensity-score matching removes some of the factors that classical migration theories suggest as reasons for an immigrant’s mental health advantage i.e. that immigrants tend to be healthier and better educated [1]. Thus, the results from this study demonstrate that a mental health advantage exists in the immigrant compared to the non-immigrants when balancing the two groups for all observed potential confounding factors. The effect may therefore be explained by certain personality factors that the migration process may select for. A study looking at Asian migration to the US describes a selection factor in migration whereby individuals are more likely to take risks and able to negotiate difficult circumstances, a so-called “psychological hardiness” [7]. This selection effect may mean that immigrants have a more robust personality that enables them to cope with adverse factors such as social stresses and adaptation to a new environment. Another potential reason for this observed mental health advantage may be because the individuals that migrated to the UK may have come from developing and sometimes war-torn countries and had actively chosen to move to the UK to seek a better life. Settling into the UK may have provided a better quality of life which may have translated to better mental health.

In order to compare the mental health of immigrants to a more representative non-immigrant UK population, we carried out an additional analysis removing ethnic group from the propensity score. In this matched analysis, there were 711 (38.3%) White non-immigrants which was more than the main analysis, although still lower than what would reflect the true indigenous UK population. In this analysis the mental health of immigrants was still significantly better than non-immigrants, which further endorses this immigrant mental health advantage that may be explained by the selection of personality traits.

### Strengths and Limitations

The study has a number of strengths. The EMPIRIC survey contains a wealth of data that we were able to use, including the validated CIS-R measure of mental health, as well as other socio-demographic and health factors. By carrying out a propensity-score matched analysis, we were able to balance several characteristics of the participants mitigating bias due to confounding, this enabled us to compare the effects of immigration status on mental health. We also tested for the influence of unmeasured confounders and demonstrated the results to be resilient to overestimation or positive selection bias.

There are also some limitations to the study. Firstly, the propensity-score matching did not produce entirely adequately balanced groups to the stated <20% standardised difference; social class III (non-manual) had a standardised difference of 21% (4.16% non-immigrants, 9.24% immigrants). However, the remaining social class categories were balanced and it is unlikely that this 1% imbalance affected the outcomes. The EMPIRIC data sample was collected in 2000 which provides a relatively dated sample, given the vast changes in the UK population in the last 15 years the sample is not representative of the current UK immigrant population. This is particularly evident by the lack of White immigrants in this dataset; the current UK immigrant population has many more European migrants than 15 years ago e.g. Polish migrants. Another limitation is the cross-sectional aspect of the data used; this meant we did not have longitudinal measures such as multiple mental health assessments with increased duration of residence or the mental health of participants prior to migration. The present study also examines immigrant status as an isolated independent variable; different immigrant subgroups may are likely to demonstrate great heterogeneity and future studies should consider this.

### Clinical Implications

This present study suggests that there may be a selection factor for immigrants which provides them with more robust personality traits and “psychological hardiness”. If circumstances arise where the mental health of these immigrants deteriorate, which may be due to prolonged exposure to the susceptibility factors this population face, there is the potential that psychological management strategies such as counselling and cognitive-behavioural strategies may be particularly effective due to possessing these personality traits. Therefore, funding and resources to provide these services in the immigrant’s native language, if necessary, may be worthwhile. However further studies need to explore the effectiveness of such support services for mental health in immigrants to establish whether this population would particularly benefit.

## Conclusions

By using propensity-score matched analysis, the findings from this study showed that immigrants were associated with better mental health than non-immigrants, in certain ethnic minority groups in the UK. Future studies should look at confirming this effect in Caucasian immigrant groups and also look at the effects of length of residence in the UK on mental health outcomes in immigrants.
